# A sensitive colorimetric probe for detection of the phosphate ion

**DOI:** 10.1038/s41598-020-78261-x

**Published:** 2020-12-04

**Authors:** Yin-Chien Chen, Kuang-Min Lo, Yu-Xian Wang, Tai-Chia Chiu, Cho-Chun Hu

**Affiliations:** Department of Applied Science, National Taitung University, 369, Sec. 2, University Rd., Taitung, Taiwan

**Keywords:** Environmental sciences, Chemistry, Nanoscience and technology

## Abstract

In the present article, we report a novel colorimetric probe (TNT@MB) for the detection of the phosphate ion, which is based on the strong binding affinity between the phosphate ion and titanium dioxide nanotubes (TNTs). TNTs were synthesized from TiO_2_ nanoparticles by hydrothermal treatment. The obtained TNTs had an average length of 200 ± 50 nm and an average width of 12 ± 5 nm. TNT@MB was prepared by adsorbing methyl blue onto TNTs in acidic condition. The optimal synthesis conditions for TNT@MB consisted in having 0.05 g of TNTs react with 1 μmole of methyl blue at pH 2 for 90 min. TNTs and TNT@MB were characterized by UV–vis diffuse reflection spectroscopy, TEM, FTIR, and XPS. The phosphate-ion sensing behavior of TNT@MB was investigated by UV–visible spectroscopy. The phosphate-ion concentration linear range and detection limit of this method based on TNT@MB were 1–40 μM and 0.59 μM, respectively. A sample of lake water was used as a real sample, and analyte recovery rates were measured in the 102.5–103.6% range, with relative standard deviations below 5.6% (n = 3). We also found that this probe could be reused after regeneration in alkaline solution. These results indicate that as a colorimetric probe, TNT@MB has the advantages of being environmentally friendly, inexpensive, and simple to use, as well as giving rise to an easily observable color change.

## Introduction

The phosphate ion is a necessary nutrient for the growth of most organisms, and it is a key chemical component of nucleic acids, proteins, and molecular energy carriers^1,2^. The phosphate ion is also very important in modern agriculture and industry. Fertilizers, animal feed, detergents, foods, and even drinks contain many phosphates^3,4^. The widespread use of phosphates leads to the production of large amounts of phosphorus-containing wastewater. Phosphorus-containing wastewater discharges, on the other hand, have a serious negative effect on the environmental quality of water bodies. The most common problem associated with phosphate ion pollution is eutrophication^5^. Therefore, it is very important to develop a simple phosphate ion sensor. Many approaches exist to detecting phosphates, such as those relying on colorimetry, chromatography, fluorometry, electrochemistry, and enzyme-based biosensing^6–11^. However, implementing most of these methods requires laborious analytical methods, the use of expensive instruments, and the synthesis of complex compounds.

In recent years, nanosensors for the detection of the phosphate ion have been extensively developed. For instance, fluorescent carbon dots (C-dots) synthesized by hydrothermal treatment of potatoes have been used to detect the phosphate ion based on the fact that C-dot fluorescence can be quenched efficiently by Fe^3+^, but it can be recovered through the addition of phosphate^8^. Single-layered graphene quantum dots (s-GQDs) have also been used to detect the phosphate ion via a similar strategy. Specifically, the aggregation-induced emission enhancement (AIEE) effect between Al^3+^ and s-GQDs is associated with an enhancement of s-GQDs' fluorescence. The strong coordination of the phosphate ion to Al^3+^, however, results in a decrease in the AIEE effect and quenching of s-GQDs fluorescence^12^. Silver nanoclusters (AgNC)/metal–organic shell composites were successfully prepared for the detection of the phosphate ion. These species were obtained via the deposition on the coordination shell around AgNCs of metal–organic (zinc–nitrogen) species that had been synthesized starting from zinc nitrate and imidazole-2-carboxaldehyde. The AgNCs/metal–organic shell composite is destroyed in the presence of the phosphate ion, due to this ion’s high affinity for Zn^2+^, resulting in a change in intensity of the fluorescence of the composite^13^.

When compared with fluorescence-based analytical approaches, colorimetry is relatively easy to employ, as it relies on color changes that may be visible with the naked eye. For example, a colorimetric assay based on the reaction competition between carboxylate group-modified gold nanoparticles (AuNPs) and Eu was used to measure phosphate concentration^6^. In another study, novel gold nanoparticles (MPTP-Zn-AuNPs) were synthesized based on the reaction between citrate-stabilized AuNPs and the 4′-(4 mercaptophenyl)-2, 2′:6′,2″-terpyridine zinc complex (MPTP-Zn). The observed color change of the MPTP-Zn-AuNPs induced by the presence of the phosphate ion was attributed to the affinity between the phosphate ion and Zn^2+^^14^. Notably, the most commonly used colorimetric method for the detection of the phosphate ion is the traditional molybdenum blue method. This approach is based on a series of chemical reactions between ammonium molybdate and the phosphate ion. However, ammonium molybdate is an expensive and potentially harmful chemical, so the development of an environmentally friendly and cheap method for the detection of the phosphate ion in solution remains an important research goal.

Titanium dioxide (TiO_2_) is a non-toxic, biocompatible, and low-cost compound^15^. Recent studies demonstrated that TiO_2_ has a good phosphate-ion sorption capacity^16–18^. In addition, TiO_2_ nanotubes (TNTs) were proven to have a larger specific surface area than TiO_2_ nanoparticles (P25)^19^. Furthermore, in acidic solution, methyl blue (MB) has been reported to be adsorbed effectively by metal oxides via the MB’s sulfonate groups^20,21^. In the present study, a colorimetric probe dubbed TNT@MB was successfully synthesized via the reaction of MB with TNTs, and the optimal phosphate ion detection conditions for the probe thus developed were investigated. To demonstrate the practicality of this system, we utilized it in the determination of the concentration of the phosphate ion in real water samples.

## Experimental

### Reagents and materials

Titanium dioxide (P25) was purchased from UniRegion Bio-Tech (Taiwan). Methyl blue (MB), Sodium phosphate monobasic dihydrate (NaH_2_PO_3_·2H_2_O), adenosine triphosphate (ATP), adenosine diphosphate (ADP) and adenosine monophosphate (AMP) were purchased from Sigma-Aldrich (USA). The lake water was collected from Jinsin Lake in the campus of National Taitung University which was selected as the real water sample. All chemicals used in the experiments were analytical grades and used as received. Ultrapure water was obtained from Milli-Q purification system.

### Instrumentation

UV–vis absorption spectra were performed on U-2900 UV–vis absorption spectrophotometer (Hitachi, Japan) and UV-2700 UV–vis diffuse reflectance spectroscopy (UV-DRS) (Shimadzu, Japan). Transmission electron microscopy (TEM) images was collected by a JEM-2100 transmission electronic microscope (Hitachi, Japan). X-ray photoelectron spectroscopy (XPS) were carried out on a XPS spectrometer (K-Alpha, Thermo, USA). FTIR spectrum were conducted on a Fourier Transform Infrared spectroscopy (Perkin Elmer, USA). The zeta potential of materials were analyzed using a Zetasizer Nano-ZS90 dynamic light-scattering (DLS) analyzer (Malvern, UK).

### Synthesis of titanium dioxide nanotube

Titanium dioxide nanotube (TNT) was synthesized by hydrothermal method modified from the earlier work^22^. Typically, 0.5 g P25 was added to 20 mL NaOH (10 M), and the mixture was transferred to stainless steel autoclave and heated at 130 °C for 20 h. After cooled down to room temperature, the precipitate were separated by centrifugation and rinsed with HCl (0.1 M) for several times. The washed precipitates were immersed in HCl (0.1 M) solution for 12 h and washed with distilled water. At last, the resultant samples were dried at 80 °C for further use.

### Preparation of TNT@MB

0.5 g titanium dioxide nanotube (TNT) was added to 10 mL Methyl blue (1 mM) (MB) and pH was adjusted to 2 by HCl (0.1 M). After stirring 90 min, the precipitates were dried at 80 °C to obtain a dark blue powder. The synthesized TNT@MB was kept in dark place before use.

### UV–vis measurements

The detection of phosphate was performed in acetic acid buffer. 5 mL different concentrations of phosphate (400–10 μM), 5 mL buffer (0.2 M, pH = 4.3) and 40 mL ultrapure water were mixed together in centrifuge tube. Afterwards, 0.05 g TNT@MB was added into tube and kept shaking for 20 min. About 3 mL of solution was take out and centrifuged at 12,000 rpm for 5 min to collected supernatant which was detected by UV–vis absorption spectrum. The detection of ATP, ADP and AMP was used same experimental procedures as above. All processes were conducted at room temperature.

### Regeneration and reuse of probe

In order to study the regeneration and reuse of probe, the TNT@MB was cleaned by alkaline solution. Three consecutive adsorption/regeneration cycles were performed. After TNT@MB was reacted with phosphate, the precipitate was collected by centrifugation. Then the precipitate was desorbed with 200 mL of 0.1 M NaOH solution and stirred for 30 min. After each cycle of desorption, the regenerated TNT was washed with ultrapure water until neutral pH and dried at 80 °C. Finally, the methyl blue was adsorbed again, and then the TNT@MB was dried for reuse in the next cycle.

### The determination of phosphate in real sample

Water sample was centrifuged at 12,000 rpm for 10 min and filtered by a 0.22 μm membrane to remove large particles. After pretreatment, water sample was then spiked with various concentration of phosphate and measured by TNT@MB using same experimental procedures as above.

## Results and discussion

**Figure 1:**
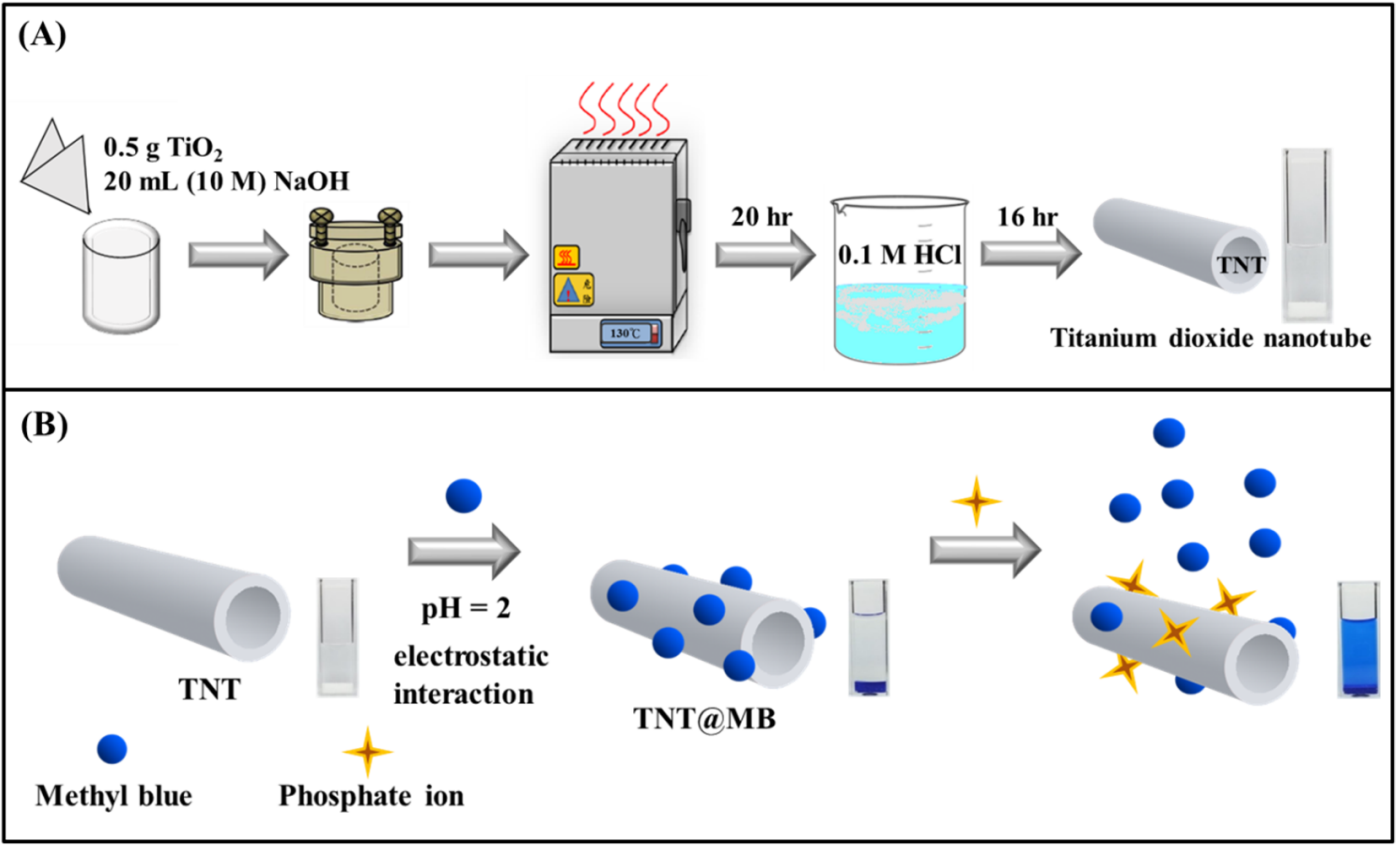
Schematic illustrations of fabrication of TNT (**A**) and TNT@MB composite for detection of phosphate (**B**).

The syntheses of TNTs and TNT@MB, as well as the process, by which the phosphate ion was detected, are illustrated in Fig. 1. TNTs were prepared by hydrothermal treatment of a specific formulation of P25, and TNT@MB was prepared by adsorbing MB onto TNTs. When TNT@MB was added to a phosphate ion containing sample, MB adsorbed on the surface of TNTs was replaced by the phosphate ion, released into solution, and detected by instrument or even naked eye.

### Investigation of the synthetic conditions and characterization of TNTs and TNT@MB

**Figure 2:**
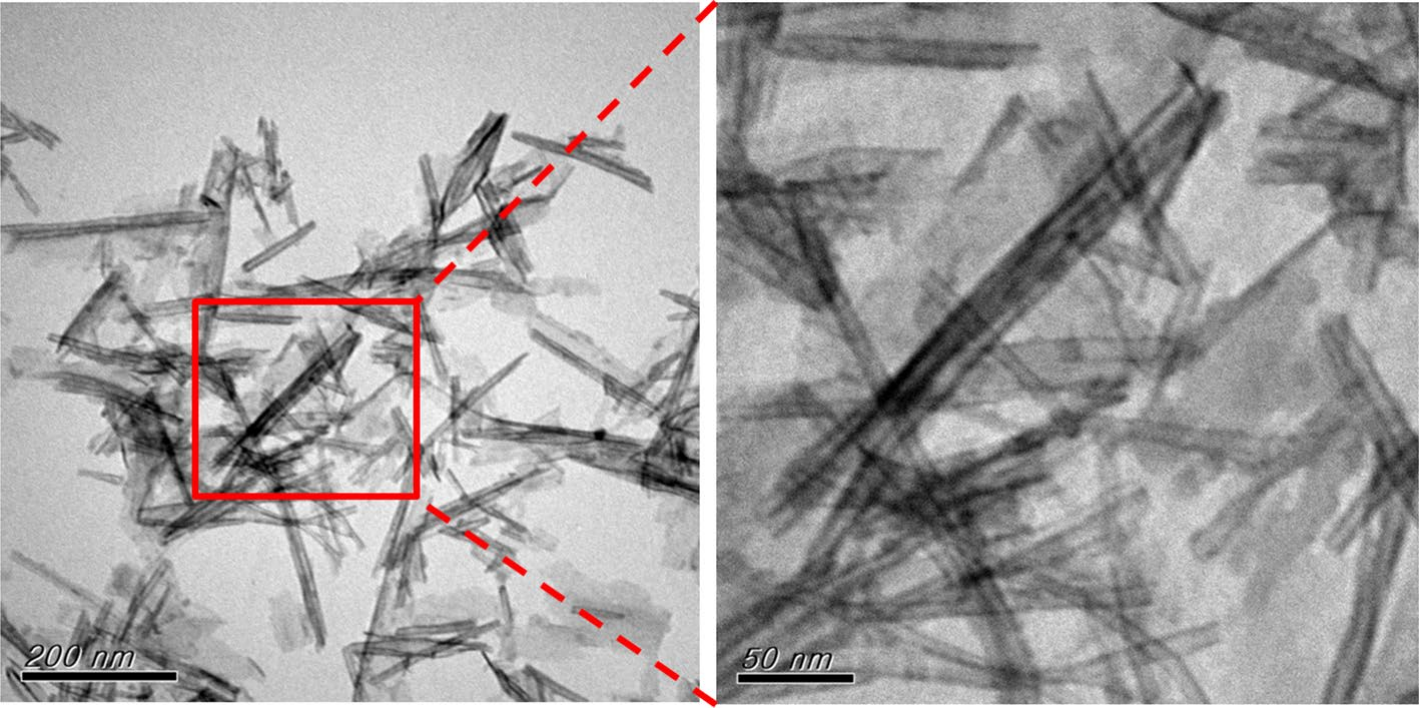
TEM images of TNT.

We compared the adsorption behavior of P25 and TNTs with that of MB. The results reported in Fig. S1 indicates that MB could be completely adsorbed by both P25 and TNTs. As depicted in the photography in Fig. S1A, P25 had smaller specific gravity than TNTs. As a result, P25 was suspended in solution, making any change of color more difficult to observe with the naked eye. TEM images of TNTs and TNT@MB are reported in Fig. 2 and Fig. S2. These images reveal that TNTs have hollow and tubular structures with length in the 150–200 nm range as well as width in 12 ± 5 nm. These results indicated that P25 could be converted into a tubular structure by the implementation of a hydrothermal method. This conclusion was similar to that reached in previous studies^23,24^. The surface zeta potentials of TNTs as a function of the pH of the solution are presented in Fig. S3. Evidence indicates that the zeta potentials of TNTs decreased monotonically as the pH increased, due to the progressive deprotonation of surface hydroxyl groups. The pH in conditions of zero overall charge (pH_pzc_) of the TNTs was ~ 4. This evidence suggests that the surface of TNTs was positively charged at pH values below 4 and that under this pH threshold an electrostatic attraction existed between MB and the surface of TNTs. Therefore, the adsorption of MB onto TNTs in acidic conditions can be interpreted as resulting from an electrostatic interaction.

In the effort to evaluate the effect of pH on the adsorption efficiency of MB onto TNTs, adsorption experiments were conducted at pH values ranging between 2 and 6. As can be evinced from the data reported in Fig. S4, adsorption efficiency increased abruptly when the solution’s pH decreased from 6 to 2. This increase in adsorption efficiency can be attributed to charge changes on the surface of the adsorbent, which would result in an increase in the attractive force between the positively charged adsorbent and the anionic adsorbate. In particular, in acidic conditions, MB would present negatively charged sulfonate groups, which would be strongly electrostatically attracted by the positively charged groups on the surface of adsorbent TNTs. Evidence indicates that at pH 2, MB was adsorbed onto TNTs almost completely. Therefore, the syntheses of TNT@MB were carried out at this pH.

**Figure 3:**
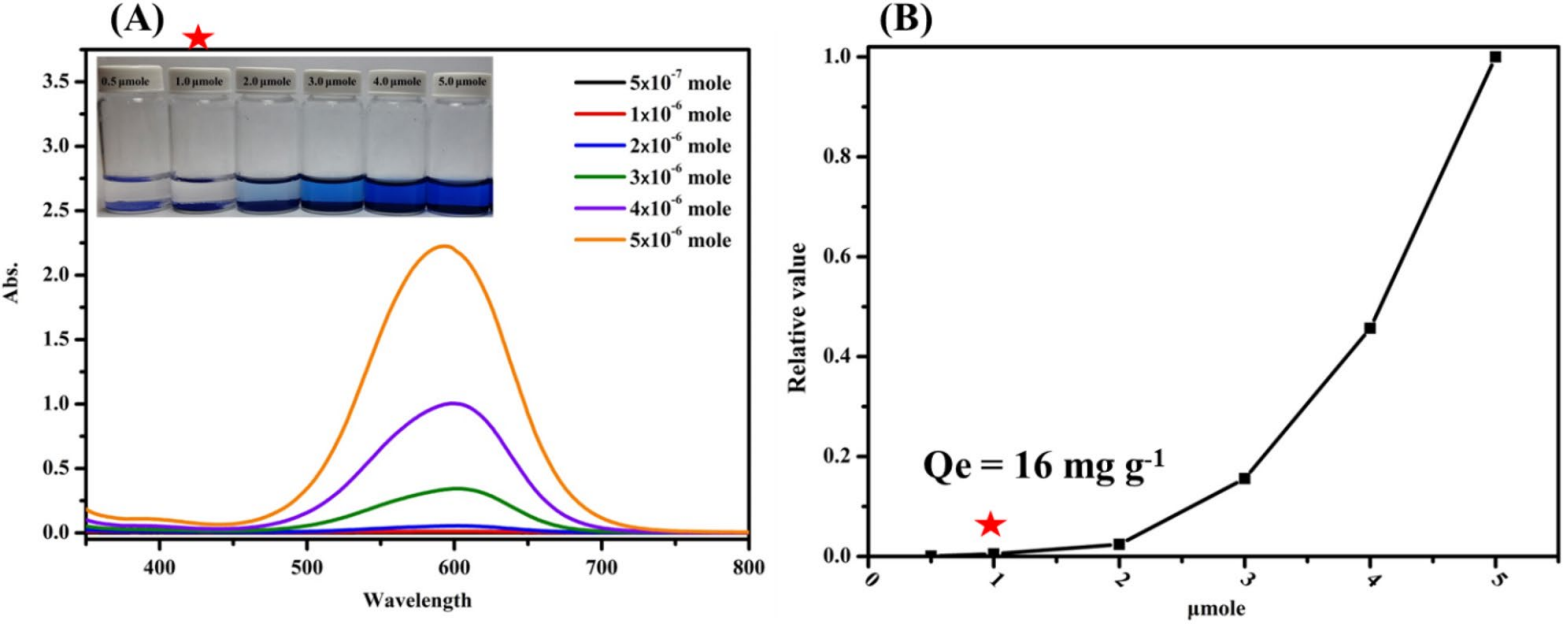
UV–vis spectra (**A**) and curve(**B**) of MB residue of 0.05 g TNT adsorbed different mole MB (10 mL).

The adsorption behavior of TNTs (0.05 g) with varying amounts of MB were then investigated. The UV–vis spectra of MB residues in solution are reported in Fig. 3. The optimal adsorption was observed when 0.05 g of TNTs were made to react with 1 μmole of MB (equilibrium adsorption amount Qe = 16 mg g^−1^). The adsorption time was then investigated making 1 μmole of MB (10 mL) react with 0.05 g of TNTs. Results from this experiment are reported in Fig. S5. In the adsorption of MB on TNTs, the reaction equilibrium was reached within 60 min. To ensure complete adsorption, 90 min was thus chosen as the optimal adsorption time.

**Figure 4:**
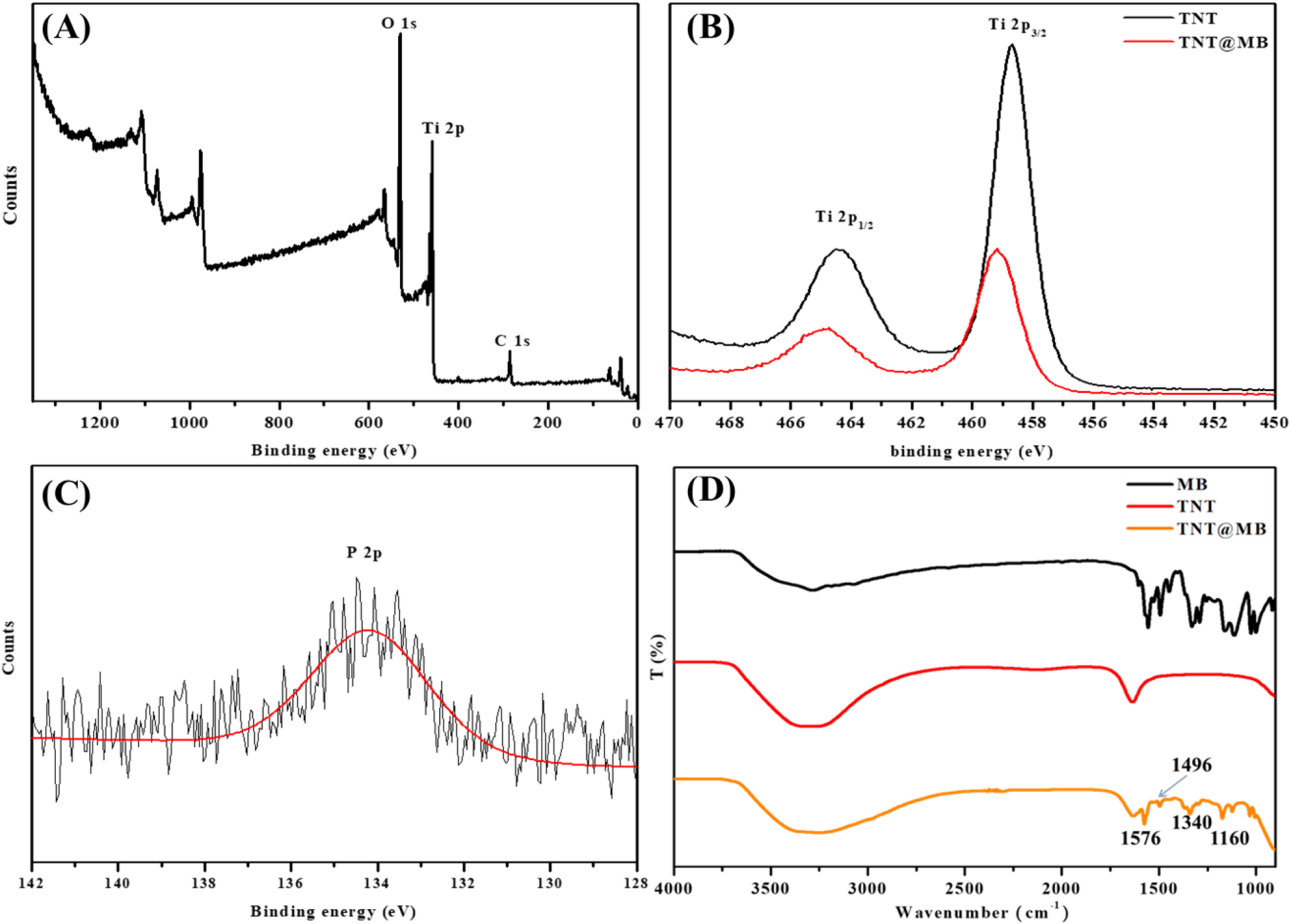
Survey XPS spectrum of TNT@MB (**A**), Ti 2p of TNT(black line) and TNT@MB(red line) (**B**), P 2p of TNT@MB incubation with phosphate (**C**). FT-IR spectrum of MB, TNT and TNT@MB (**D**).

The UV–vis absorption spectra of TNTs and TNT@MB are reported in Fig. S6. TNTs displayed a strong absorption in the 300–350 nm range, which is attributable to the band-gap of TiO_2_^25^. TNT@MB exhibited a wide and strong absorption in the 500–700 nm visible range, alongside a decrease in intensity of the absorption due to the band-gap of TiO_2_. Therefore, TNTs have the appearance of a white-colored powder, whereas TNT@MB is a dark blue powder. The XPS survey spectrum of TNT@MB is reported in Fig. 4A. Found in this spectrum are the peaks associated with Ti, C, and O. Figure 4B presents the Ti 2p spectra of TNTs and TNT@MB. In the case of the unreacted TNTs, the peaks at 458.68 eV and 464.48 eV could be attributed to Ti 2p_3/2_ and Ti 2p_1/2_, respectively. The energy difference between these two peaks (5.8 eV) indicates that titanium was in its Ti(IV) oxidation state^26,27^. After MB was adsorbed onto TNTs, the Ti(IV) oxidation state of TNTs shifted to higher binding energies, and the above-mentioned peaks shifted to 459.18 eV (Ti 2p_3/2_) and 464.93 eV (Ti 2p_1/2_). These results suggest that, upon formation of TNT@MB, the chemical environment of Ti in TNTs has changed. The P 2p spectrum of TNT@MB incubated with the phosphate ion is reported in Fig. 4C. In this case, the presence of a peak at 133.5 eV confirmed the successful incorporation of phosphate on TNTs^28^. The FTIR spectra of TNTs and TNT@MB are reported in Fig. 4D. The broad bands in the 3100–3500 cm^−1^ range and the band at 1630 cm^−1^ were separately assigned to the stretching and bending vibration of the O–H functional group belonging to water molecules adsorbed on the surface of TNTs^17^. The peaks at 1340 cm^−1^ and 1160 cm^−1^ are due to the stretching vibrations of the S=O group, whereas the peaks at 1576 cm^−1^ and 1496 cm^−1^ are due to the stretching vibrations of the C=C and C=N groups, respectively^29^. The location and appearance of these signals are in conformity with those expected for the characteristic functional groups of MB. Therefore, these spectral evidence confirmed that MB can be adsorbed on the surface of TNTs.

### Detection of the phosphate ion

We presumed that the behavior of TNT@MB in the presence of the phosphate ion descended from the fact that the phosphate ion has a stronger tendency to adsorb on the surface of TNTs than that of MB. In order to confirm this hypothesis, we compared the affinity for TNTs of the phosphate ion to that of MB (Fig. S7). For this purpose, the 0.05 g of TNTs were added to a solution that contained only MB at 15 μM concentration, and the resulting supernatant was collected after centrifugation. The supernatant was detected by UV spectrometry (Fig. S7 red line). Results indicated that MB had been adsorbed by the TNTs, leading to a decrease in the intensity of the absorption peak at 600 nm. The black line visible in Figure S7 represented the experiment whereby the same amount of TNTs were added into a mixed solution of 15 μM MB and 10 μM phosphate ion. This approach had the advantage of forcing the phosphate ion to compete with MB for the binding sites on the surface of TNTs. Results of this proof-of-concept experiment indicated the possibility of using MB-coated TNTs to detect phosphate ions directly.

**Figure 5:**
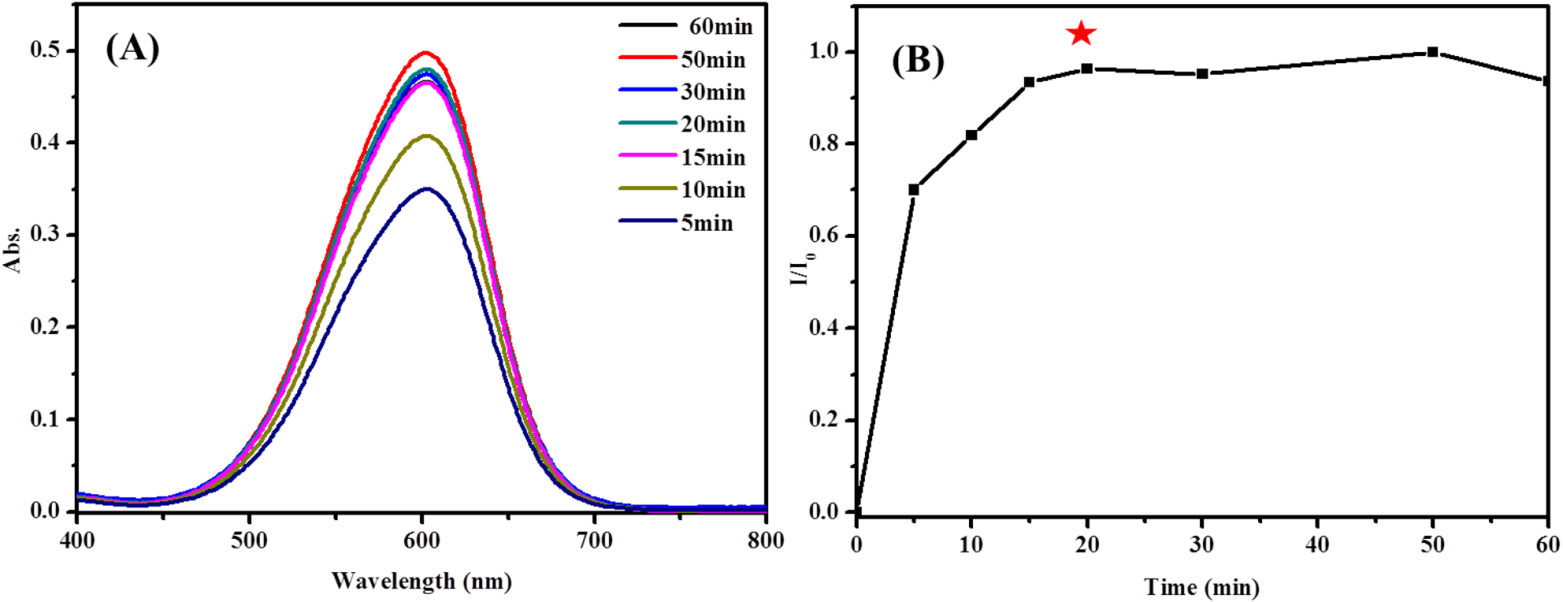
UV–vis spectra of time-dependent MB desorption amount (**A**) and desorbed capacities (**B**) of 0.05 g TNT@MB in 0.1 mM phosphate solution (10 mL).

**Figure 6:**
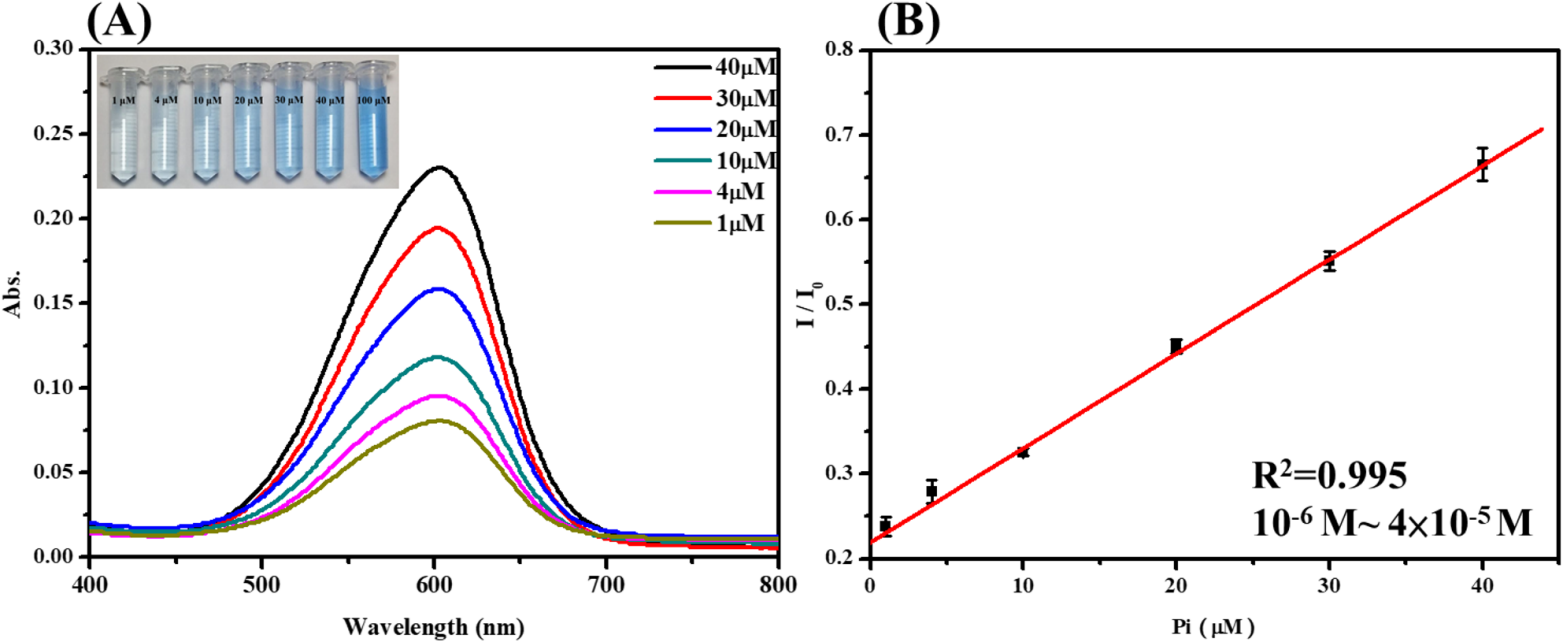
UV–vis spectra of MB desorption amount (**A**) and plot of the absorption intensity versus the different concentrations phosphate ion added (**B**). Where I_0_ was the absorption intensity of TNT@MB with 0.1 mM phosphate. Condition: in buffer solution (pH 4.3).

On the basis of the data reported in Fig. 5, it could be observed that, following addition of the phosphate ion to TNT@MB, the absorption intensity of MB increased from 1 to 20 min, whereas not much change was evident between 20 and 60 min. These data suggest that the reaction between TNT@MB and the phosphate ion was complete within 20 min. The UV–vis spectra of the systems containing TNT@MB and the phosphate ion at different concentrations are reported in Fig. 6A. As can be evinced from the data in Fig. 6B, absorption intensity increased alongside the concentration of the phosphate ion. In particular, the absorption intensity increased linearly with the phosphate ion concentration in the 1–40 μM range (R^2^ = 0.995). Additionally, the limit of detection (LOD) of the phosphate ion by the TNT@MB system was estimated to be 0.59 μM.

**Figure 7:**
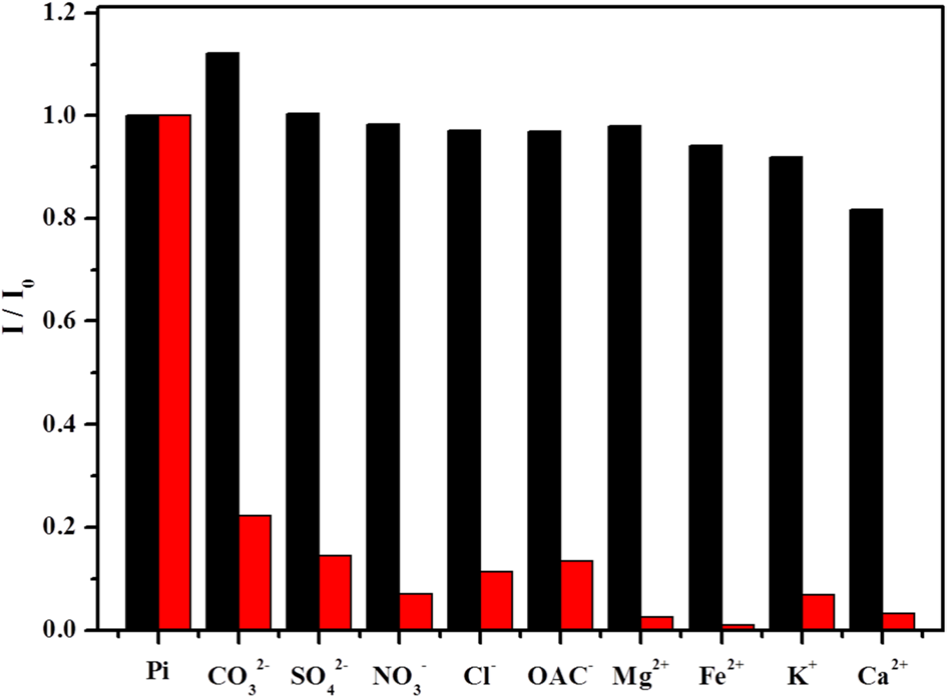
Selective adsorption response of TNT@MB (red column) towards phosphate ion (10^–4^ M) and interference (black column) of different ions (10^–4^ M).

The specificity of this novel colorimetric probe was then investigated by adding to the TNT@MB system various potentially interfering substances, which could be present in natural water samples, substances, in particular, containing ions like CO_3_^2−^, SO_4_^2−^, NO^3−^, Cl^−^, Hg^2+^, OAc^−^, Ag^+^, Mg^2+^, K^+^, and Ca^2+^. Notably, only small changes were detected in the absorption spectrum of the TNT@MB system in the presence of these anions and metallic cations. (Fig. 7) In the presence of carbonate, the solution’s pH increased, which caused MB to be easily desorbed into solution as a consequence of the weakening of the electrostatic attraction between the negatively charged surface of TNTs and MB. The phosphate ion LOD and linear range of our system and those of other methods used for phosphate ion detection are reported in Table S1. Results indicate that the LOD and linear range for TNT@MB as a probe for the phosphate ion are acceptable from the standpoint of environmental sensing. It should be noted, furthermore, that TNT@MB is more eco-friendly, cheaper, and characterized by a more clearly detectable color change than other nanosensors reported previously.

### Phosphate ion detection in a real sample and regeneration of the sensor

We used TNT@MB to estimate the phosphate ion level in a water sample collected from a lake. We spiked lake water samples with standard PO_4_^3−^ solutions (10 μM and 15 μM). The results of the recovery tests (values obtained from 102.5% to 103.6%) were satisfying, and the relative standard deviations were less than 5.6% (Table 1). The phosphate ion concentration detected was in the 1–40 μM range (R^2^ = 0.992), and the LOD was 0.68 μM.

**Table 1:** Analytical results of phosphate in lake water sample.

Spiked concentration (M)	Recovered concentration (M)	Recovery (%) (n = 3)	RSD (%)
0	1.89 × 10^–5^	–	–
1.0 × 10^–5^	2.93 × 10^–5^	103.6	1.4
1.5 × 10^–5^	3.43 × 10^–5^	102.5	5.6

**Figure 8:**
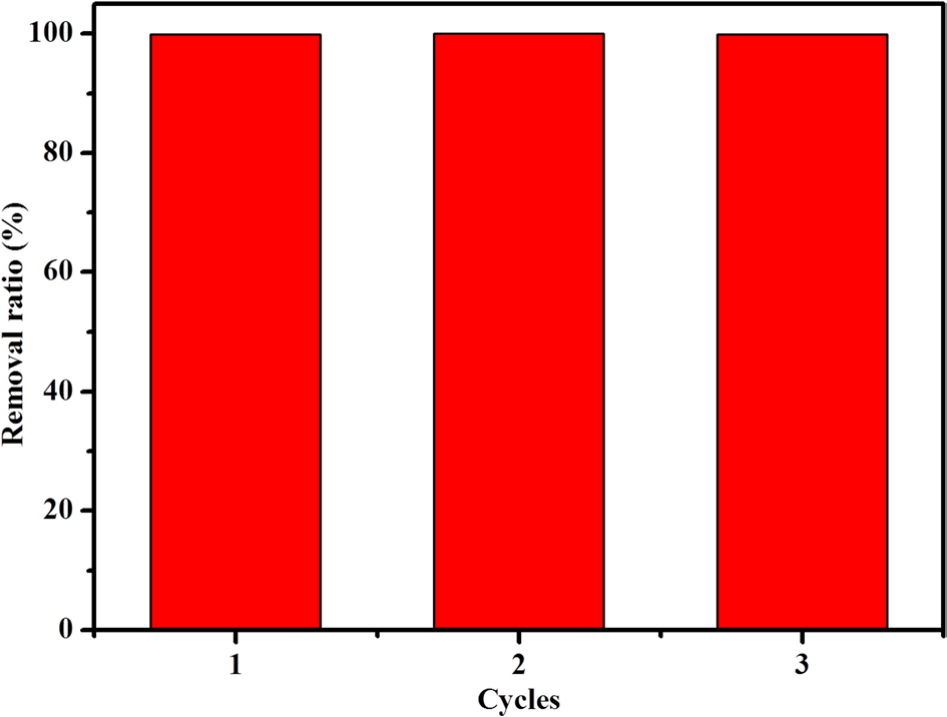
Removal ratio of MB by TNT at different regeneration cycles (readsorption conditions: initial MB concentration 1 mM, 10 mL at pH 2.0, adsorbent dose 0.05 g).

The effective regeneration and reuse of a phosphate ion probe is of crucial importance for the probe’s large-scale application, so the reusability of TNT@MB was examined over three adsorption–desorption cycles. The data on the regeneration of TNT@MB as performed by its treatment with a 0.1 M NaOH solution followed by reaction between the NaOH-treated TNTs and MB as “Preparation of TNT@MB” section described, to achieve re-adsorption onto TNTs are reported in Fig. 8. Results indicate that the NaOH-treated TNTs maintain almost 100% MB adsorption efficiency even after three cycles, suggesting that TNT@MB is characterized by excellent recyclability.

Additionally, we also used TNT@MB to detect ATP, ADP, and AMP (Fig. S8). Results indicate that this colorimetric probe had a higher sensitivity for detecting ATP than ADP or AMP. Evidence thus suggests that triphosphate-containing molecules like ATP interact more strongly with TNTs than do their diphosphate-containing and monophosphate-containing counterparts. The following experiments were continuous processed. We believe this colorimetric probe also has a good potential as a biomedical sensor.

## Conclusion

In summary, a new and sensitive colorimetric probe for the detection of the phosphate ion was developed based on the modification of TNTs with MB. The optimal synthetic and sensing conditions for this sensor were investigated. This colorimetric probe enabled us to detect the phosphate ion based on the strong binding affinity between the phosphate ion and TNTs, with the mutual binding between these species made evident by a color change detectable with the naked eye. In addition, this probe can be reused after being washed with an alkaline solution. Results from the present study indicate that the colorimetric probe developed has the advantages of being environmentally friendly, inexpensive, and easy to reuse. It also has the key advantage of giving rise to a color change that can be easily observed with the naked eye.

## Supplementary Information

Supplementary Information
