# Supplementary material for: A sensitive colorimetric probe for detection of the phosphate ion
Source: Sci Rep. 2020 Dec 4;10:21215. doi: 10.1038/s41598-020-78261-x (PMC7718235; doi:10.1038/s41598-020-78261-x)
Supplement: Supplementary file 1 — Supplementary Information [file 41598_2020_78261_MOESM1_ESM.docx]

*Electronic Supplementary Information*

**A sensitive colorimetric probe for detection of the phosphate ion**

Yin-Chien Chen^a^, Kuang-Min Lo^a^, Yu-Xian Wang^a^, Tai-Chia Chiu^a,b^ and Cho-Chun Hu^a,b^*

^a^ Department of Applied Science, National Taitung University,

^b^ Agriculture Products Inspection Centre, National Taitung University

369, Sec. 2, University Rd., Taitung, Taiwan, R.O.C.

**Yin-Chien Chen**: jj24ka@gmail.com

Kuang-Min Lo: alan112601@gmail.com

Yu-Xian Wang: yoci50155@gmail.com

Tai-Chia Chiu: tcchiu@nttu.edu.tw

Cho-Chun Hu: cchu@nttu.edu.tw


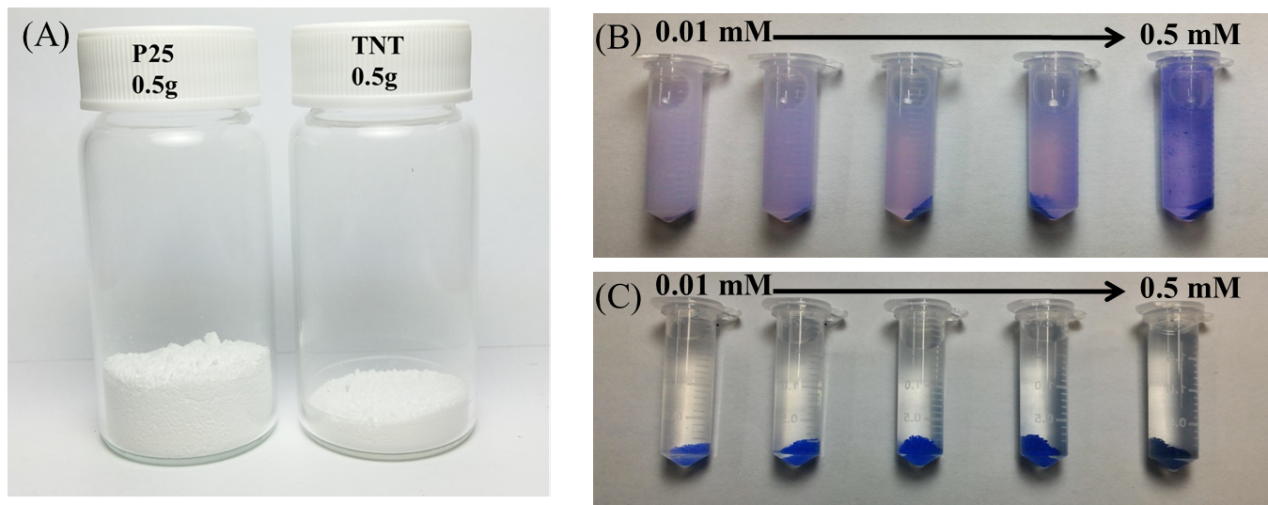


**Fig. S1.** Photos of P25 and TNT (A).Different concentrations MB (0.01, 0.03, 0.05, 0.1, 0.5 mM) were adsorbed by P25 (B) TNT (C) for 2 hours.


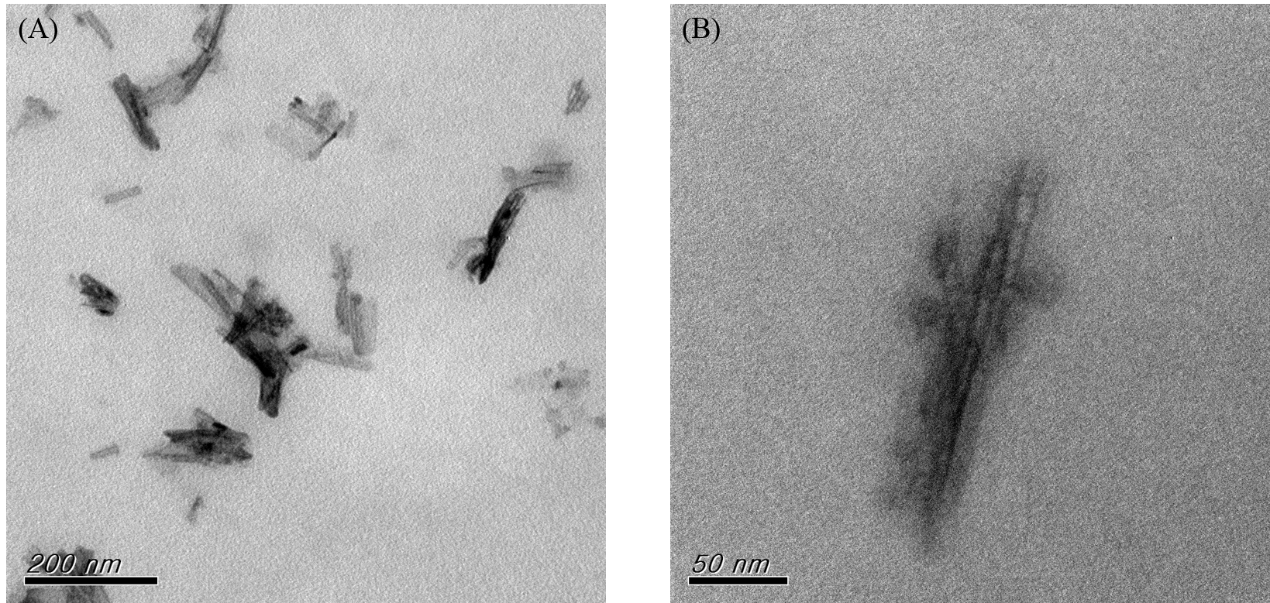


**Fig. S2.** TEM images of TNT@MB.


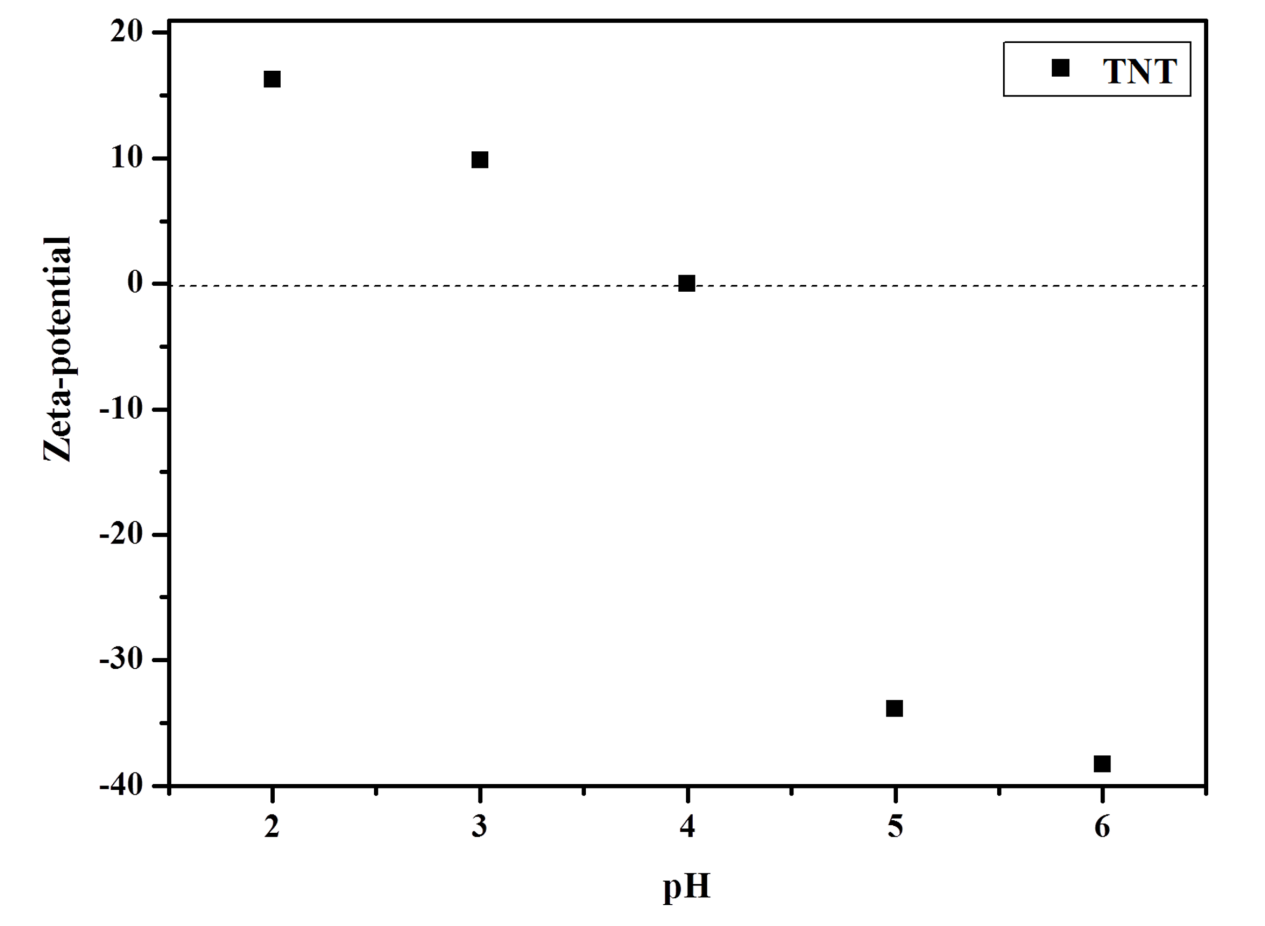


**Fig. S3.** Zeta-potential of TNT at different pH.


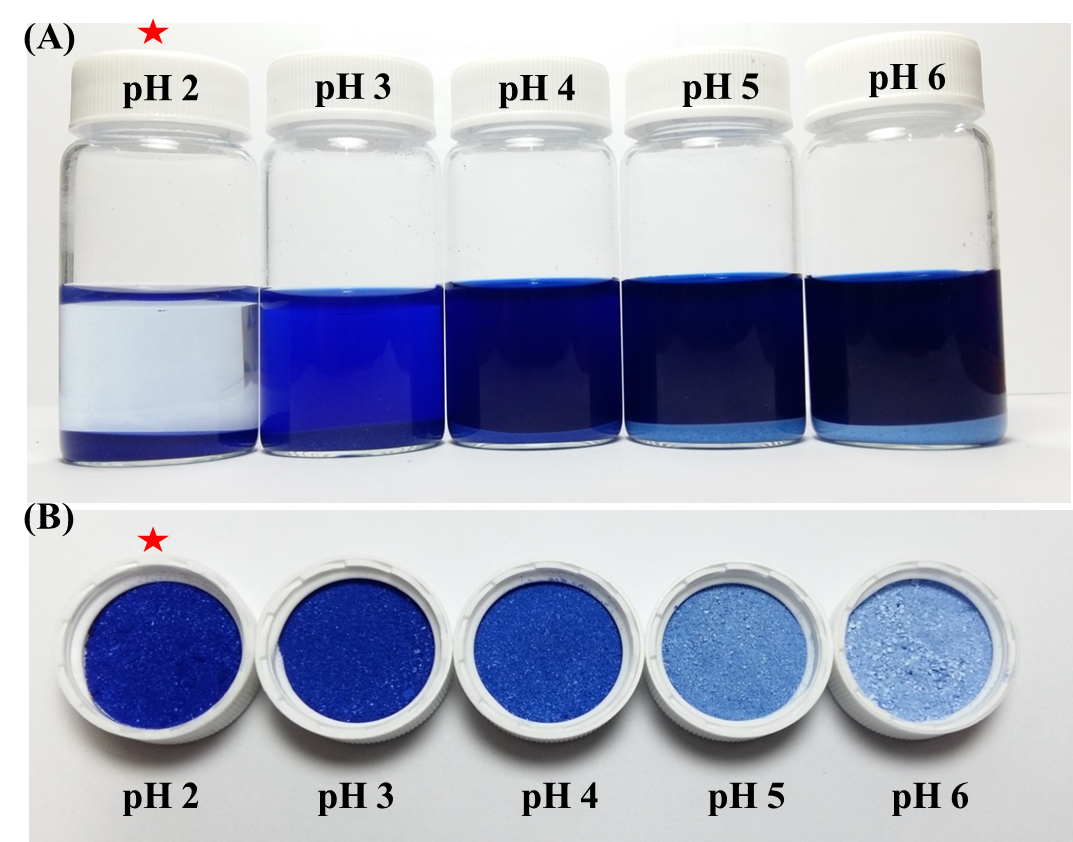


**Fig. S4*.*** Photograph of MB adsorbed on TNT (A) and dried powder of TNT@ MB(B) in different pH.


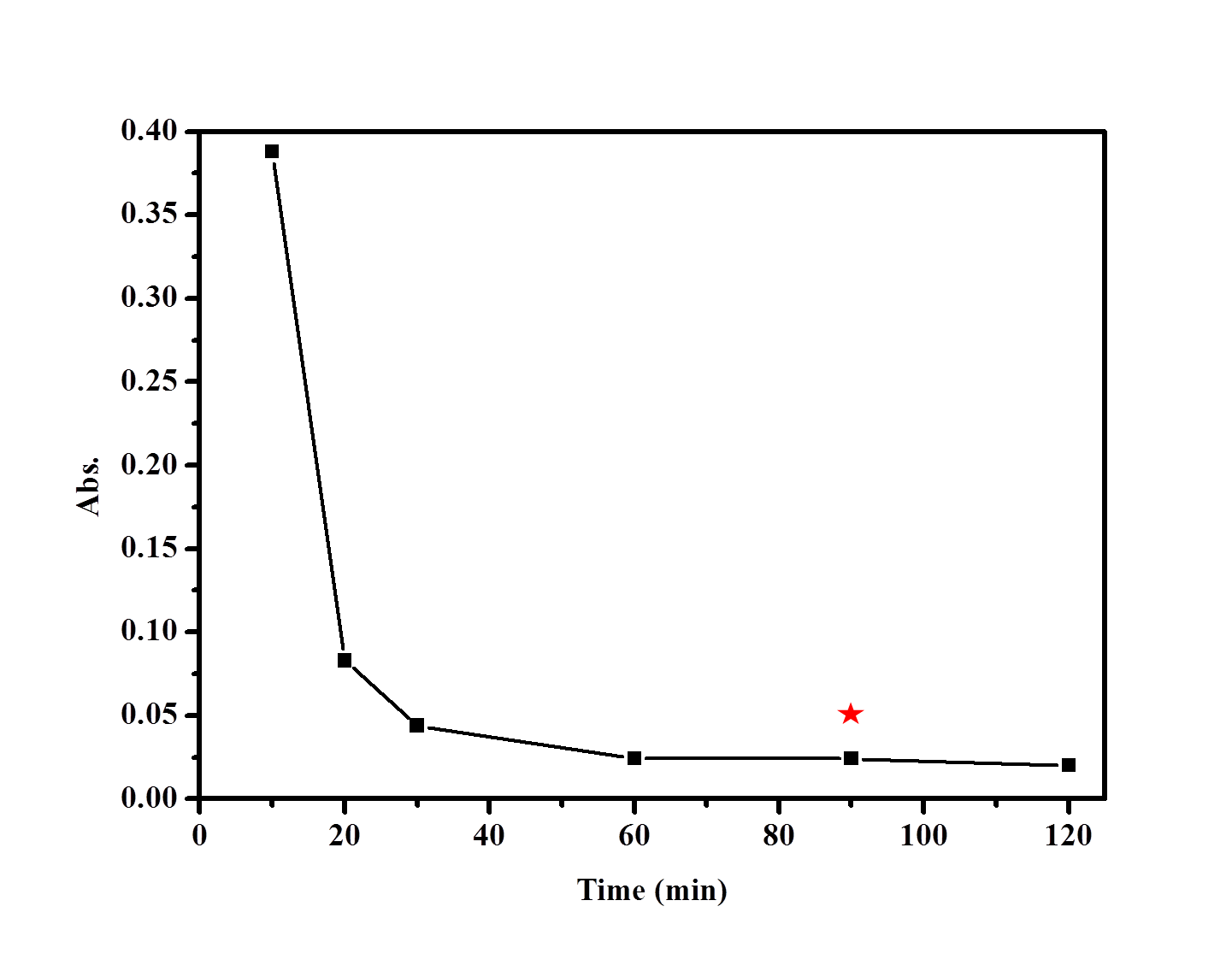


**Fig. S5*.*** Time-dependent MB residue of 1 μmole MB (10 mL) adsorbed by 0.05 g TNT.


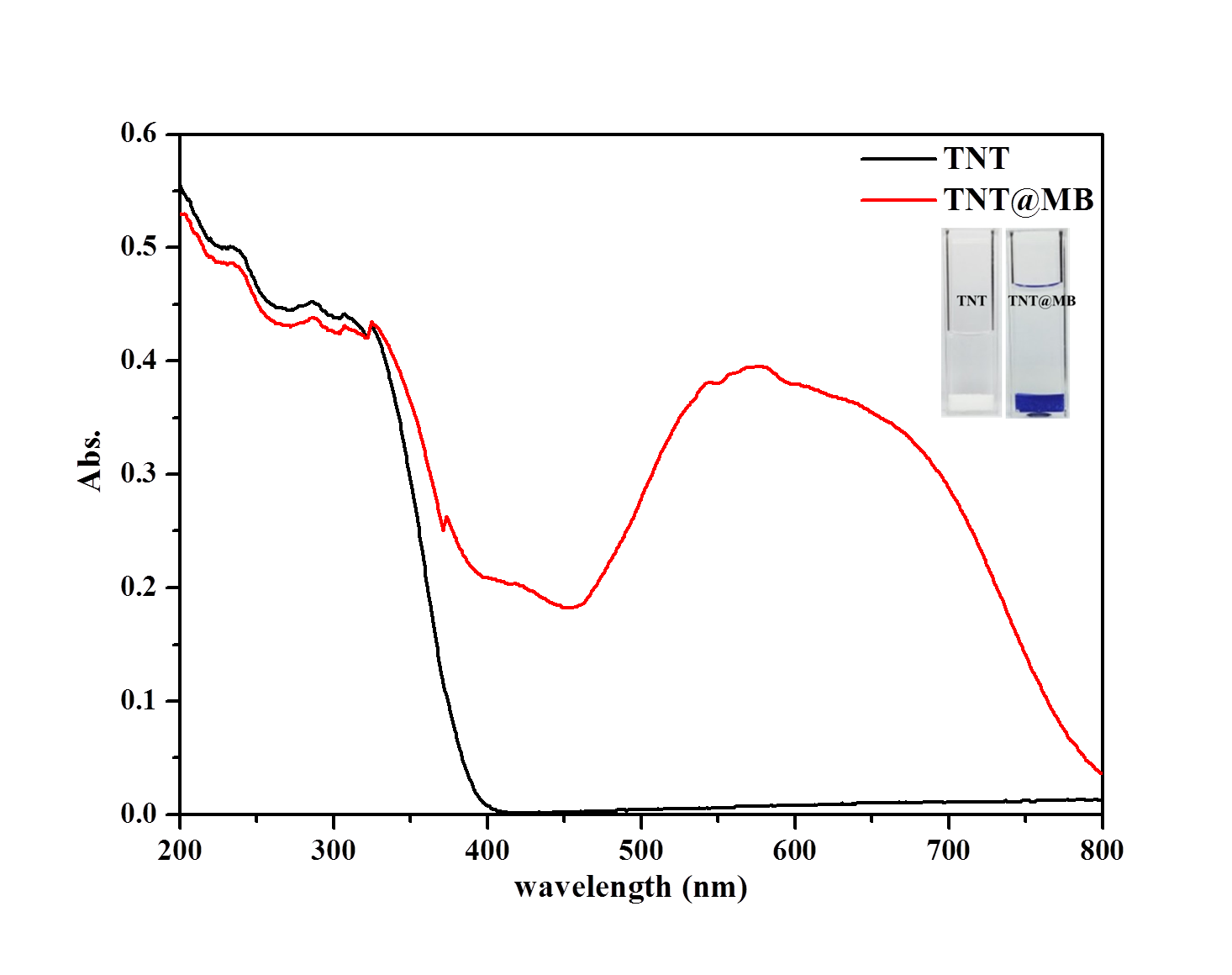


**Fig. S6.** UV–vis diffuse reflection spectra of TNT (black line) and TNT@MB (red line).


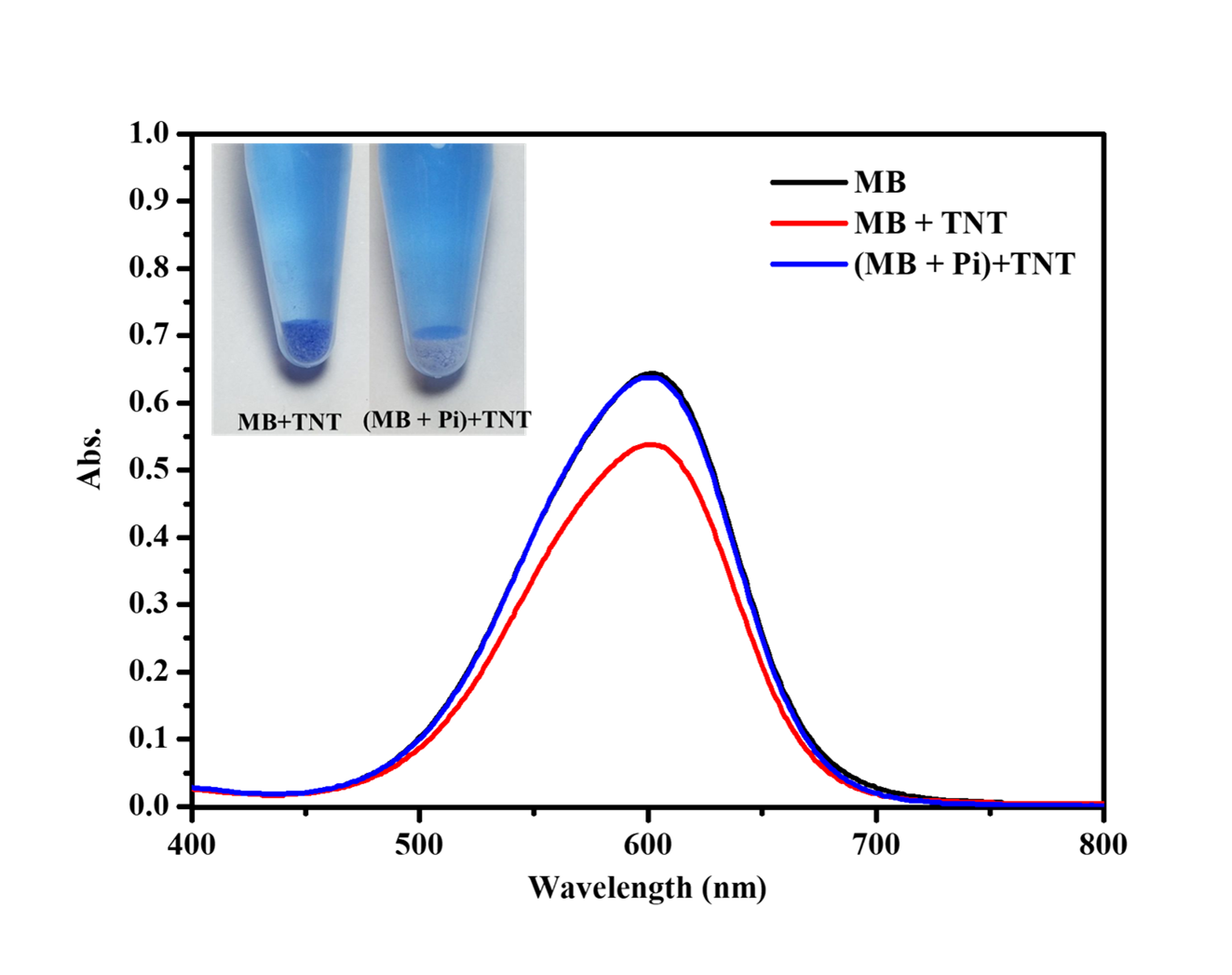


**Fig. S7.** UV-vis spectra of 15 μM MB (black line), 0.05 g TNT mixed with 15 μM MB (red line) and 0.05 g TNT mixed with 15 μM MB containing 10 μM phosphate (blue line) in buffer solution (pH 4.3).


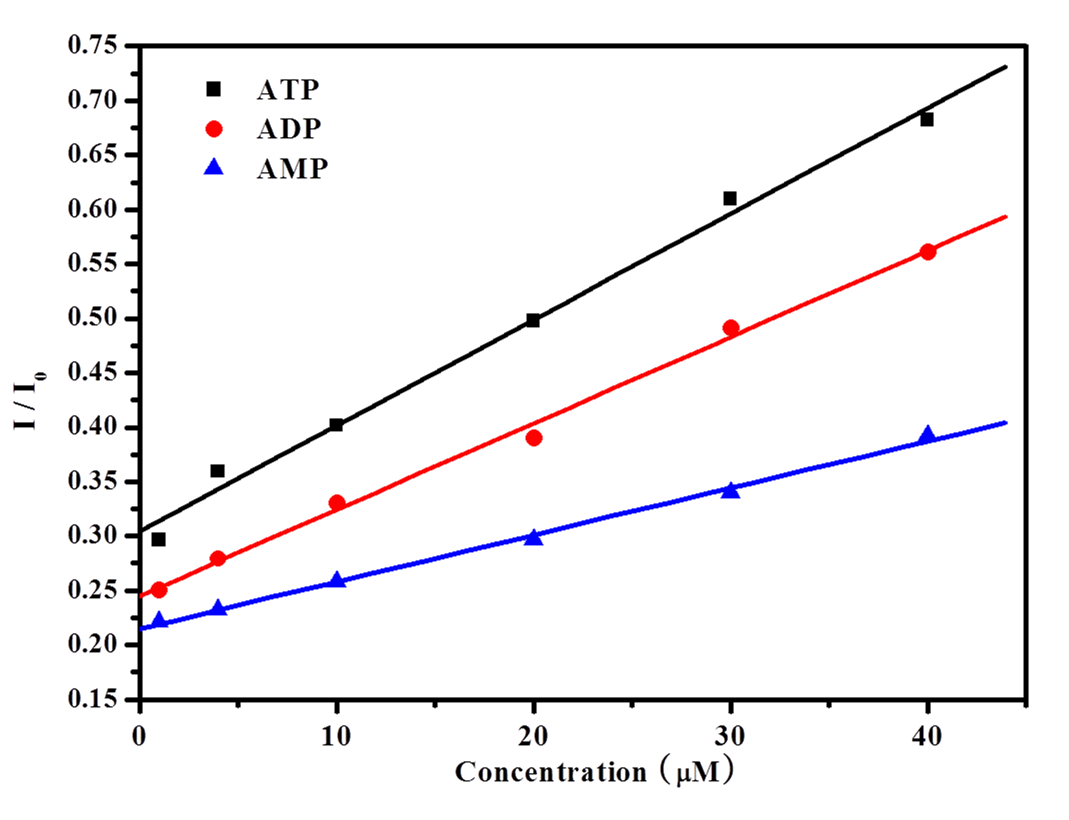


**Fig. S8.** MB desorbed amount in the presence of various concentrations of ATP, ADP and AMP. Where I_0_ was the absorption intensity of TNT@MB with 0.1 mM phosphate. Condition: in buffer solution (pH 4.3).

| **Table S1.** Comparison of the analytic performance of different phosphate sensors. | | | | |
| --- | --- | --- | --- | --- |
| Method | Materials | Liner Range | LOD | Ref. |
| fluorescent | Eu-ICP | 2$-$100 μM | 0.83 μM | [^1^](#_ENREF_1) |
| fluorescent | TGA capped Cd TeQDs | 0.2$-$2.4 mM | - | [^2^](#_ENREF_2) |
| fluorescent | RBMB@CNPs | 0.5$-$50 µM | 0.08 µM | [^3^](#_ENREF_3) |
| fluorescent | Carbon-dots with Fe^3+^ | 10$-$250 µM | 0.80 µM | [^4^](#_ENREF_4) |
| fluorescent | AgNCs/metal−organic shell | 1$-$100 µM | 0.06 µM | [^5^](#_ENREF_5) |
| fluorescent | Ag_2_SQDs/metal-  oragnic shell | 0.7$-$88.2 µM | 70.00 nM | [^6^](#_ENREF_6) |
| absorption | MPTP-Zn-AuNPs | 80$-$200µM | 11 ppm | [^7^](#_ENREF_7) |
| absorption | MA-AuNPs | 0.5$-$30µM | 76.00 nM | [^8^](#_ENREF_8) |
| absorption | Fe_3_O_4_ MNPs | 0.2$-$200µM | 0.11 μM | [^9^](#_ENREF_9) |
| absorption | TNT@MB | 1$-$40 µM | 0.59 µM | This work |

ICP is infinite coordination polymer.

TGA is thioglycolic acid.

RB is rhodamine B, MB is methylene blue and CNPs is Coordination Nanoparticles.

Ag NCs is silver nanoclusters.

Ag_2_S QDs is Ag_2_S quantum dots.

AUNPs is gold nanoparticles

MPTP is 4′-(4-mercaptophenyl)-2,2′:6′,2″-terpyridine

MA is mercaptoacetic acid

MNPs is magnetite nanoparticles

1 Song, X. *et al.* Europium-based infinite coordination polymer nanospheres as an effective fluorescence probe for phosphate sensing. *RSC Advances* **7**, 8661-8669 (2017).

2 Borse, V., Jain, P., Sadawana, M. & Srivastava, R. ‘Turn-on’fluorescence assay for inorganic phosphate sensing. *Sensors and Actuators B: Chemical* **225**, 340-347 (2016).

3 Lin, N. *et al.* A turn-on coordination nanoparticle-based fluorescent probe for phosphate in human serum. *Nanoscale* **7**, 4971-4977 (2015).

4 Xu, J. *et al.* Carbon dots as a luminescence sensor for ultrasensitive detection of phosphate and their bioimaging properties. *Luminescence* **30**, 411-415 (2015).

5 Dai, C., Yang, C.-X. & Yan, X.-P. Ratiometric fluorescent detection of phosphate in aqueous solution based on near infrared fluorescent silver nanoclusters/metal–organic shell composite. *Analytical chemistry* **87**, 11455-11459 (2015).

6 Yan, D., He, Y., Ge, Y. & Song, G. Fluorescent Detection of Phosphate in Aqueous Solution Based on Near Infrared Emission Ag 2 S QDs/Metal− Organic Shell Composite. *Journal of fluorescence* **27**, 227-233 (2017).

7 He, G., Zhao, L., Chen, K., Liu, Y. & Zhu, H. Highly selective and sensitive gold nanoparticle-based colorimetric assay for PO43− in aqueous solution. *Talanta* **106**, 73-78 (2013).

8 Liu, W., Du, Z., Qian, Y. & Li, F. A specific colorimetric probe for phosphate detection based on anti-aggregation of gold nanoparticles. *Sensors and Actuators B: Chemical* **176**, 927-931 (2013).

9 Chen, C. *et al.* A new colorimetric protocol for selective detection of phosphate based on the inhibition of peroxidase-like activity of magnetite nanoparticles. *Analytical Methods* **7**, 161-167 (2015).
